# Attention-deficit/hyperactivity disorder associated with KChIP1 rs1541665 in Kv channels accessory proteins

**DOI:** 10.1371/journal.pone.0188678

**Published:** 2017-11-27

**Authors:** Fang-Fen Yuan, Xue Gu, Xin Huang, Yu-Wei Hou, Yan Zhong, Jun Lin, Jing Wu

**Affiliations:** 1 Department of Epidemiology and Biostatistics and the Ministry of Education (MOE) Key Lab of Environment and Health, School of Public Health, Tongji Medical College, Huazhong University of Science and Technology, Wuhan, People’s Republic of China; 2 Department of Child Health Care, Hunan Children’s Hospital, Changsha, People’s Republic of China; 3 Department of rehabilitation medicine, Wuhan Medical and Health Center for Women and Children, Wuhan, People’s Republic of China; Kunming Institute of Zoology, Chinese Academy of Sciences, CHINA

## Abstract

Attention-deficit/hyperactivity disorder (ADHD) is an early onset childhood neurodevelopmental disorder with high heritability. A number of genetic risk factors and environment factors have been implicated in the pathogenesis of ADHD. Genes encoding for subtypes of voltage-dependent K channels (Kv) and accessory proteins to these channels have been identified in genome-wide association studies (GWAS) of ADHD. We conducted a two-stage case–control study to investigate the associations between five key genes (*KChIP4*, *KChIP1*, *DPP10*, *FHIT*, *and KCNC1*) and the risk of developing ADHD. In the discovery stage comprising 256 cases and 372 controls, *KChIP1* rs1541665 and *FHIT* rs3772475 were identified; they were further genotyped in the validation stage containing 328cases and 431 controls.*KChIP1* rs1541665 showed significant association with a risk of ADHD at both stages, with CC vs TT odds ratio (OR) = 1.961, 95% confidence interval (CI) = 1.366–2.497, in combined analyses (*P*-FDR = 0.007). Moreover, we also found rs1541665 involvement in ADHD-I subtype (OR (95% CI) = 2.341(1.713, 3.282), and Hyperactive index score (*P* = 0.005) in combined samples.Intriguingly, gene-environmental interactions analysis consistently revealed the potential interactionsof rs1541665 collaboratingwith maternal stress pregnancy (*P*_mul_ = 0.021) and blood lead (*P*_add_ = 0.017) to modify ADHD risk. In conclusion, the current study provides evidence that genetic variants of Kv accessory proteins may contribute to the susceptibility of ADHD.Further studies with different ethnicitiesare warranted to produce definitive conclusions.

## Introduction

Attention-deficit/hyperactivity disorder (ADHD) is an early onset childhood neurodevelopmental disorder with a prevalence estimated at 5.9%-7.1% worldwide [1, 2]. ADHD is diagnosed approximately three times more frequently in boys than in girls [3]. Core characteristics of the disorder are pervasive and developmentally inappropriate inattention, excessive motor activity, impulsivity, and distractibility [4]. Sufferers usually have marked social, academic, occupational and family difficulties, and approximately 30%-50% of them have persisting symptoms as adults [5, 6]. Furthermore, a diagnosis of ADHD conveys a significant risk for other comorbid diseases, such as conduct disorder, learning disorders, mood disorders, and anxiety disorders [6, 7].

The etiology of ADHD remains unclear. Family studies reported that ADHD heredity is as high as 80%–90%, indicating a strong genetic component [6]. In addition to genetic factors, environmental factors involving in exposure to adverse circumstances in maternal or children lifehave been implicated in theetiology of ADHD [8]. Studies addressing gene-environment interactions mayplay a pivotal role in ADHD [9]. Great effort has been put into seeking potential genes conferring susceptibility to ADHD. In the first genome-wide association studies (GWAS) of ADHD performed by Neale et al., the top 25 single-nucleotide polymorphisms (SNPs) (based on *P*-value) implicated as interesting candidate genes included two genes encoding for voltage-gated potassium (Kv) channel-interacting protein 1 (KChIP1) and Kv channel-interacting protein 4 (KChIP4) [10]. At the same time, Lesch et al. observed that a potassium channel (KCNC1) gene was among the top 30 hit genes for association analyses of the ADHD diagnostic phenotype [11]. GWAS analysis conducted by Lasky-Su et al. found genes (*KChIP1* [alias name *KCNIP1*], *KChIP4* [alias name *KCNIP4*], dipeptidyl-peptidase 10 [*DPP10*], and fragile histidine triad protein [*FHIT*]) related to potassium-channel function associated with total ADHD symptom count [12]. In addition, in an ADHD pathway analysis, pathways containing Kv channels genes have been the most significant [13]. Furthermore, candidate gene based association studies in adult ADHD patients have suggested a role of *KChIP4* gene in ADHD susceptibility [14].

Kv channel genes have been classified as subfamiliesShaker (*Kv1*.*1* to *1*.*8*), *Shab* (*Kv2*.*1* and*Kv2*.*2*), *Shaw* (*Kv3*.*1* to *3*.*4*), and *Shal* (*Kv4*.*1* to *Kv4*.*3*)according to *Drosophila* genes and regulated by many accessory proteins including Kv channel-interacting proteins (KChIPs) and dipeptidyl peptidase-like proteins (DPLPs,). *KChIP4*, *KChIP1* and *DPP10* have been found binding to subunits of the Kv4.2 family to modulate their cell-surface expression and subcellular localization [15, 16]. KChIPs causes a dramatic redistribution of Kv4.2, allowing for trafficking to the cell surface and decrease in the degradation of enhanced stability Kv4.2 through KChIPs binding [17, 18]. KChIP co-expression also causes phosphorylation to reconstitute the molecular properties of Kv4.2 [19]. In addition, DPP10facilitated Kv4.2 protein trafficking to the cell membrane, increased A-type current magnitude [20].

Kv channels and accessory proteinsplay important roles in cellular signaling processes and are critical to neurotransmission [21]. Pharmacological activation of Kv channels in excitable cells reduces excitability, whereas channel inhibition has the opposite effect of increasing excitability [22]. It is believed that neurotransmission and neuronal excitability play an important role in the pathogenesis of ADHD [23, 24] Genetic studies also supported the role of Kv channels in the regulation of dopaminergic transmission [25, 26]. Given that impairments in axonal dopamine release are associated with ADHD [27], we speculated about the possibility that Kv channels may have a role in ADHD etiology.

To date, no combined analysis for the genes of Kv channels and ancillary proteins to these channels in ADHD has been performed. Therefore, we carried out a two-stage case–control study to comprehensively evaluate the associations between five key genes (*KChIP4*, *KChIP1*, *DPP10*, *FHIT*, *and KCNC1*)and the risk of ADHD. Also, given the etiological complexity of ADHD, it was deemed important toinvestigate the interactions between these genes andenvironmental risk factors implicated in ADHD.

## Materials and methods

### Study subjects

We applied a two-stage case-control study to investigate the association of genetic variants inKv channels and accessory proteins with ADHD risk. The discovery sample (stage one) included 256 ADHD children and adolescents with ADHD consecutively recruited from Wuhan Medical and Health Center for Women and Children between October 2013 and December 2014, and 372 control children from the same hospital who underwent physical examination during the same time period. The validation sample (stage two) included 328 ADHD cases enrolled from the Children's Hospital of Hunan province (Changsha) from January 2014 to December 2015, and 431 controls selected among children who visited the same hospital for physical examination during the same time period. All cases were diagnosed ADHD for the first time according to the diagnostic criteria of the DSM-IV [1]; Clinical interviews of at least one parent were conducted by a trained child psychiatrist. According to DSM-IV, we determined subtypes of ADHD cases as follows: combined (ADHD/C), predominantly inattentive (ADHD/I) and predominantly hyperactive/impulsive (ADHD/HI) [28, 29].

All subjects were required to meet the following criteria: (1) be between 6 and 18 years old; (2) have a full scale Intelligence Quotient (IQ≥70) according to the Chinese-Wechsler Intelligence Scale for Children [30]; (3) children with neurological disorders, seizure disorders, pervasive developmental disorders, bipolar mood disorders, or psychotic disorders were excluded.

This study was approved by the Ethics Committees of Tongji Medical College of Huazhong University of Science and Technology, Wuhan Medical and Health Center for Womenand Children and Children's Hospital of Hunan province. At recruitment, written informed consent was obtained from the parents of each subject, and blood samples and demographic information were collected.

### Measurement of environment factors

Information about basic demographic variables (age, gender, IQ) and other potential risk factors for ADHD was obtained from the questionnaire, including maternal stress pregnancy (The total score>0 was coded yes, otherwise, no), maternal smoking or alcohol pregnancy (If the mother smoked or drinkedat any time during the pregnancy, the mother was coded yes, otherwise, no), parental marital status (couple or divorced), preterm birth (<37 weeksor ≥37 weeks), low birth weight (<2.5kg or ≥2.5kg) and blood lead.

To measure maternal stress pregnancy, the 30-item Chinese version of Pregnancy Stress Rating Scale (PSRS) was used [31, 32]. The total score is the mean of all items summed, with higher scores indicating higher maternal stress. 0 means the mother experiences no stress; 0.001–1 means the mother experiences a mild level of stress; 1.001–2 means the mother experiences a moderate level of stress; and 2.001–3 means the mother experiences a severe level of stress.

To measure lead level in blood, 3–5mL of whole blood was drawn from each child and was collected in heparin-containing tubes. Blood lead level (BLL)was determined by atomic absorption spectrophotometry (AA-670/GV-5, Shimadzu, Japan) at a commercial laboratory. The coefficient of variation for the BLLs was 4.9%. The limit of detection for blood lead was 0.2μg/dL. None of the blood samples below the limit of detection are shown. In our study, a median was used for cutoff point for differentiating blood lead level; thus, median or more was defined as indicative of a high lead level and less than median of a low lead level.

### Conners Parent Symptom Questionnaire (PSQ)

ADHD symptoms were measured with a Chinese version of the Conners Parent Symptom Questionnaire (PSQ) [33, 34]. The PSQ contains 48 items and 6 subscales (Conduct problem, Difficulties in learning, Psychosomatic disorders, Hyperactivity/Impulsivity, Anxiety and Hyperactivity index). Parents answered questions concerning their child's behavior over the past month using a four-point scale (0 = not true at all to 3 = very much true). Scores were converted to T scores based on the gender and age of the child, with scores >65 indicating clinically elevated symptoms [35]. The three subscale scores that focused on ADHD symptoms (Hyperactivity/Impulsivity score, Hyperactivity index score, and Total score) were used as primary outcome measures. The hyperactivity index score was mostly used to reflect the hyperactivity behavior of ADHD children, while the Hyperactivity/Impulsivity score implicated both hyperactivity and impulsivity behaviors.

### Candidate SNPs selection and genotyping

Tag SNPs were selected based on SNPs genotype information downloaded from Hapmap (http://hapmap.ncbi.nlm.nih.gov/, HapMap Data Rel24/phaseII Nov08, on NCBI B36 assembly, dbSNP b126) for the CHB (Han Chinese from Beijing) population, using the criteria of r^2^>0.8 and minor allele frequency (MAF) >0.15 across the region of the candidate genes. We placed the selected tag SNPs into an integrated bioinformatics tool “F-SNP” (http://compbio.cs.queensu.ca/F-SNP/) [36] and retrieved a set of functionally predicted SNPs with F-score>0.101, including the possible functions of splicing, transcription, translation, and post-translation processes. Additionally, those variants referred in GWAS analyses (*KChIP4* rs876477, *KChIP1* rs1541665, *DPP10* rs272000, and *FHIT* rs6791644) were also included in our study. Finally, 17 SNPs were identified as the candidate SNPs (S1 Table).

Genomic DNA was extracted from 2 ml of peripheral blood sample using the Relax Gene Blood DNA System DP319-02 (Tiangen, Beijing, China) according to the manufacturer’s instructions. Candidate SNPs were genotyped using MassARRAY technology (Sequenom Inc., Dan Diego, CA, USA) in both stage one and stage two according to the manufacturer’s iPLEX Application Guide. The primers were designed using the Assay Design 3.0 software supplied by Sequenom. The iPLEX^™^ reaction products were dispensed onto a 384-well SpectroChip, and they were processed and analyzed in a Compact Mass Spectrometer using the Mass ARRAY Workstation 4.0 software (Sequenom Inc., San Diego, CA, USA).

Quality control was performed by exclusion of SNPs with a genotype call rate of <90% and those that deviated from the Hardy-Weinberg Equilibrium (HWE) in controls. Notably, genotyping was performed by experimenters blinded to the status of the participants.

### Statistical analysis

The HWE for genotypes in the control groups was assessed by a *χ*^*2*^ goodness-of-fit test. Pearson’s *χ*^*2*^test or two independent sample *t*-test was used to examine differences between cases and controls in the distribution of demographic characteristics. The risks of ADHD, ADHD subtypes and CPT results associated with SNPs were estimated by using odds ratios (ORs) and the 95% confidence intervals (95% CIs) were calculated by multivariate logistic regression model (LR) after adjustment for age and gender. The Bonferroni [37] and false discovery rate (FDR) [38] methods were performed for multiple comparison corrections for association analyses. The association of Conners Parents Symptom scores with SNPs was explored by ANOVA analysis with post hoc comparisons using the Student-Newman-Keuls (SNK) method. The statistical power before the study performed in both stages was calculated using PowerV3.0 [39]. For SNPwith MAF = 0.18 in CHB, we calculated that with our sample size the power to detect an OR of 1.50 is as follows: stage one power = 0.702; stage two power = 0.782. The FDR for each SNP was estimated using the R (version 3.1.3; http://www.r-project.org/). All other statistical analyses were conducted using IBM SPSS software (version 22.0; SPSS Inc., Chicago, IL, USA).

Furthermore, to assess the high-order gene-environment interactions, multifactor dimensionality reduction (MDR) analyses were carried out using MDR 2.0 beta 8.1 program (UPenn, Philadelphia, PA, USA)[40] in combined samples. In brief, this program first constructed all possible combinations of included variables. Then, by using 10-time cross-validation and 1000-time permutation tests, the best factor models for predicting ADHD risk were found with the maximal cross-validation consistency (CVC) and the optimal testing accuracy. Finally, for these best n-factor models, the interactions were measured by logistic regression under multiplicative and additive interaction models [41, 42]. Amultiplicative interaction term was evaluated by alikelihoodratiotestintheLRmodelusingSPSSsoftwarev20.0. Departurefromadditiveinteractionwasassessed by a bootstrapping test of goodness-of-fit using STATA(version 10.0).

## Results

### Subjects characteristics

The distributions of selected characteristics in the two stages are listed in Table 1. No statistically significant difference was found between cases and controls in the distribution of age, gender and IQ score in the two stages (stage one: *P*_*age*_ *=* 0.148, *P*_*gender*_ = 0.570, *P*_*IQ*_ = 0.159; stage two: *P*_*age*_ *=* 0.266, *P*_*gender*_ = 0.076, *P*_*IQ*_ = 0.083). In stage one, we also found significant differences in the distribution of maternal stress pregnancy, parental marital status, low birth weight and blood lead level (*P* = 0.006, *P* = 0.033, *P* = 0.018 and *P* = 0.009 respectively). In stage two, significant difference was found in the distribution of maternal stress pregnancy (*P* = 0.030). For ADHD subtypes, ADHD-I was the most in both stage one (40%) and stage two (42%). For PSQ scores, significant differences have been found in the two stages for the Hyperactivity index score (stage one: *P* = 0.009; stage two: *P* = 0.001).

**Table 1:** Characteristic data of the subjects.

	Stage one				Stage two			
	ADHD	Control	Statistics	*P* value	ADHD	Control	Statistics	*P* value
	(256)	(372)	(χ^2^ or *t*)		(328)	(431)	(χ^2^ or *t*)	
Age(mean±SD)^a^	8.20±1.53	8.34±1.72	1.048	0.148	8.51±1.78	8.60±2.10	0.624	0.266
Gender(boys: girls)			0.324	0.570			3.157	0.076
Boys	201	299			263	322		
girls	55	73			65	109		
IQ score(mean±SD) ^a^	96.57±12.35	97.52±11.25	1.000	0.159	89.33±11.21	90.49±11.60	1.385	0.083
Maternal stress pregnancy								
No	149	256	7.460	**0.006**	251	349	4.700	**0.030**
Yes	107	116			77	72		
Maternal smoking								
No	241	356	0.785	0.376	314	422	3.013	0.083
Yes	15	16			14	9		
Maternal alcohol								
No	239	359	3.299	0.069	311	415	0.969	0.325
Yes	17	13			17	16		
Parental marital status								
Couple	229	350	4.525	**0.033**	314	416	3.013	0.083
Divorced	27	22			14	12		
Preterm birth								
≥37 weeks	214	330	3.425	0.064	298	380	2.870	0.090
<37 weeks	42	42			30	51		
Low birth weight								
<2.5kg	34	28	5.643	**0.018**	35	29	3.749	0.053
≥2.5 kg	222	344			293	402		
Blood lead level^b^								
Low	117	202	6.783	**0.009**	152	228	3.205	0.073
High	149	169			176	203		
Subtype								
Inattentive(ADHD-I)	102(40%)				138(42%)			
Hyperactive/impulsive(ADHD-HI)	64(25%)				79(24%)			
Combined(ADHD-C)	90(35%)				111(34%)			
PSQ score(mean±SD) ^a^								
Impulsive-hyperactive score	1.36±1.20	1.27±1.05	-0.995	0.160	1.52±0.84	1.45±0.82	-1.153	0.125
Hyperactive score	1.32±0.41	1.24±0.42	-2.368	**0.009**	1.58±0.92	1.36±0.96	-3.184	**0.001**
Total score	38.98±17.56	37.86±11.15	-0.977	0.164	40.21±18.02	38.97±16.59	-0.983	0.163

^a^ mean ± SD, by independent *t*-test;

^b^ Stage one: low <Median 54.50ug/l, High≥54.50 ug/l; Stage two: low <Median 55.05ug/l, High≥55.05ug/l; Bold values indicated *P*< 0.05.

### Association between SNPs in candidate gene and ADHD Risk

Distribution of genotypes of the SNPs in stage one was presented in S2 Table. We selected 15 SNPs for analysis for two deviating HWE (significant level α = 0.05; α' = 0.003 after Bonferronicorrection). As shown in S3 Table, codominant, dominant, recessive and additive models were all performed for every SNP. Unfortunately, all the *P* values did not surpass the Bonferroni threshold in the association tests (significant level α = 0.05; α' = 0.00067). However, as shown in Table 2, in stage one, *KCNIP1* rs1541665 was significantly associated with ADHD under the codominant model (OR = 1.972, 95% CI = 1.197–3.223, *P-FDR* = 0.028), dominant model (OR = 1.611, 95% CI = 1.169–2.319, *P-FDR* = 0.028), and addictive model (OR = 1.633, 95% CI = 1.199–2.427, *P-FDR* = 0.028) after FDR correction. *FHIT* rs3772475 has also been observed to have significant association with ADHD under dominant model (OR = 1.572, 95% CI = 1.128–2.306, *P-FDR* = 0.028).

**Table 2:** Association between promising polymorphism genotypes and ADHD risk in all stages.

Polymorphism	genotype	Cases	Controls	χ^2^	*P* value	OR(95%CI)^a^	*P*^a^ *value*	*P-FDR*^b^
KChIP1								
**rs1541665**								
Stage one	TT	104	195	9.573	0.008	1.000		
	TC	111	138			1.502(1.051,2.145)	0.025	0.060
	CC	41	39			**1.972(1.197,3.223)**	**0.011**	**0.028**
	Dominant model					**1.611(1.169,2.319)**	**0.009**	**0.028**
	Recessive model					1.628(1.027,2.615)	0.041	0.065
	Addictive model					**1.633(1.199,2.427)**	**0.005**	**0.028**
Stage two	TT	114	191	10.088	0.006	1.000		
	TC	142	177			1.346(0.979,1.843)	0.081	0.116
	CC	72	63			**1.925(1.229,2.873)**	**0.005**	**0.028**
	Dominant model					**1.434(1.078,1.969)**	**0.016**	**0.037**
	Recessive model					**1.688(1.191,2.453)**	**0.009**	**0.028**
	Addictive model					**1.588(1.125,2.257)**	**0.014**	**0.033**
Combined	TT	218	386	19.978	4.590*10^−5^	1.000		
	TC	253	315			**1.427(1.071,1.763)**	**0.009**	**0.028**
	CC	113	102			**1.961(1.366,2.497)**	2.391*10^**−4**^	**0.007**
	Dominant model					**1.559(1.259,1.976)**	7.182*10^**−4**^	**0.012**
	Recessive model					**1.639(1.292,2.297)**	**0.004**	**0.028**
	Addictive model					**1.569(1.218,1.998)**	**0.006**	**0.028**
FHIT								
**rs3772475**								
Stage one	TT	114	205	6.958	0.031	1.000		
	TC	106	128			1.517(1.194,2.195)	0.024	0.060
	CC	36	39			1.662(1.001,2,515)	0.049	0.073
	Dominant model					**1.572(1.128,2.306)**	**0.011**	**0.028**
	Recessive model					1.459(0.823,2.341)	0.173	0.236
	Addictive model					1.557(0.910,2.537)	0.165	0.236
Stage two	TT	126	181	1.907	0.385	1.000		
	TC	110	137			1.084(0.509,1.409)	0.532	0.550
	CC	92	103			1.105(0.678,1.802)	0.245	0.313
	Dominant model					1.134(0.571,1.253)	0.720	0.720
	Recessive model					1.140(0.455,2.860)	0.112	0.168
	Addictive model					1.099(0.684,1.766)	0.327	0.363
Combined	TT	245	386	6.284	0.043	1.000		
	TC	216	265			1.312(1.039,1.698)	0.029	0.062
	CC	123	142			1.319(1.052,1.871)	0.035	0.070
	Dominant model					1.476(1.043,2.089)	0.028	0.062
	Recessive model					1.017(0.605,1.708)	0.250	0.313
	Addictive model					1.233(0.958,1.587)	0.104	0.164

^a^ All the *P* values were adjusted for age and gender.

^b^ *P* values of false discovery rate (FDR). The significant results were in bold.

The promising *KChIP1* rs1541665 and *FHIT* rs3772475 were further genotyped in the validation stage. We successfully validated the significant association between *KChIP1* rs1541665 with ADHD risk (CC vs.TT, OR = 1.925, 95% CI = 1.229–2.873, *P-FDR* = 0.028; dominant model: OR = 1.434, 95% CI = 1.078–1.969, *P-FDR* = 0.037; addictive model: OR = 1.588, 95% CI = 1.125–2.257, *P-FDR* = 0.033) (Table 2) after FDR correction. However, we didn’t find *FHIT* rs3772475significant association with ADHD in validated samples.

### Association between candidate SNPs and ADHD subtypes

In stage one (Table 3), significant association was found between ADHD-I and controls for rs1541665. Compared with TT genotype, TC+CC genotype increased ADHD-I risk (OR = 2.089, 95% CI = 1.401–3.137, *P-FDR* = 0.027) after FDR correction. In stage two, rs1541665 has been consistently associated with ADHD-I (OR = 2.178, 95% CI = 1.398–3.773, *P-FDR* = 0.027) after FDR correction. In combined samples, we replicated these results (Table 4). However, we didn’t find other snps significant association with ADHD subtypes after Bonferroni correction (S4 Table, α' = 0.0011).

**Table 3:** Correlations between promising SNPs and ADHD subtypes in both stages and combined stage.

SNP	Genotype	Control	ADHD-HI				ADHD-I				ADHD-C			
			Case	OR(95%CI)	*P*^a^	*P*^*-*^*FDR*^b^	Case	OR(95%CI)	*P*^a^	*P*^*-*^*FDR*^b^	Case	OR(95%CI)	*P*^a^	*P*^*-*^*FDR*^b^
rs1541665														
Stage one	TT	195	30	Ref			34	Ref			40	Ref		
	CT+CC	177	34	1.018(0.375,1.778)	0.612	0.648	68	**2.089(1.401,3.137)**	**0.003**	**0.027**	50	1.629(0.828,2.645)	0.168	0.444
Stage two	TT	191	37	Ref			34	Ref			43	Ref		
	CT+CC	240	42	1.287(0.799,2.057)	0.312	0.468	104	**2.178(1.398,3.773)**	**0.001**	**0.027**	68	1.377(0.876,2.141)	0.189	0.437
Combined	TT	386	67	Ref			68	Ref			83	Ref		
	CT+CC	417	76	1.231(0.834,1.815)	0.791	0.868	172	**2.341(1.713,3.282)**	8.951*10^**−6**^	1.611*10^**−4**^	118	1.464(0.952,2.259)	0.085	0.250
rs3772475														
Stage one	TT	205	32	Ref			45	Ref			37	Ref		
	TC+CC	167	32	1.581(0.895,2.102)	0.194	0.437	57	1.504(0.878,2.501)	0.128	0.384	53	1.891(1.008,2.881)	0.044	0.158
Stage two	TT	181	30	Ref			54	Ref			42	Ref		
	TC+CC	240	34	0.853(0.514,2.319)	0.712	0.712	48	0.878(0.591,1.773)	0.545	0.644	48	1.397(0.725,2.459)	0.312	0.468
Combined	TT	386	62	Ref			99	Ref			79	Ref		
	TC+CC	407	66	1.220(0.789,1.999)	0.434	0.644	105	1.111(0.763,1.419)	0.445	0.616	101	1.212(0.879,1.689)	0.249	0.468

^a^ All the *P* values were adjusted for age and gender.

^b^ *P* values of false discovery rate (FDR). The significant results were in bold.

**Table 4:** Association of promising SNPs genotypes with PSQ scores in all stages.

	Genotype	Conner score								
		Impulsive-hyperactive score			Hyperactive index score			Total score		
		Mean±SD	F	*P*^a^	Mean±SD	F	*P*^a^	Mean±SD	F	*P*^a^
KCNIP1 rs1541665										
Stage one			1.672	0.097		3.266	**0.035**		2.698	0.052
	TT	1.28±0.88		Ref	1.22±0.84		Ref	36.35±14.51		Ref
	CT	1.30±0.86		0.974	1.39±0.91		0.260	39.43±16.19		0.512
	CC	1.58±1.02		0.136	1.69±0.97		**0.038**	48.60±24.87		0.041
Stage two			2.871	0.046		4.638	0.016		0.547	0.233
	TT	1.14±0.86		Ref	1.12±0.64		Ref	37.39±17.65		Ref
	CT	1.25±0.93		0.872	1.25±0.77		0.725	39.44±15.81		0.827
	CC	1.40±0.85		0.230	1.44±0.83		**0.029**	42.50±21.38		0.864
Combined			0.240	0.312		4.780	**0.014**		0.122	0.367
	TT	1.16±0.68		Ref	1.45±0.42		Ref	39.17±18.28		Ref
	CT	1.26±0.64		0.926	1.55±0.51		0.865	38.00±17.79		0.981
	CC	1.17±0.63		1.000	1.75±0.60		**0.005**	37.36±14.57		0.943
FHIT rs3772475										
Stage one			3.127	0.041		4.639	**0.016**		0.547	0.232
	TT	1.24±0.76		Ref	1.12±0.65		Ref	37.29±17.66		Ref
	TC	1.26±0.87		0.923	1.25±0.88		0.727	39.41±15.82		0.817
	CC	1.61±0.85		0.042	1.52±0.83		**0.027**	42.51±21.37		0.875
Stage two			0.204	0.330		2.322	0.064		0.478	0.242
	TT	1.17±0.61		Ref	0.94±0.69		Ref	39.47±18.33		Ref
	TC	1.26±0.69		0.894	1.28±0.86		0.481	36.92±16.75		0.737
	CC	1.20±0.69		1.000	1.39±0.75		**0.046**	36.87±12.81		0.880
Combined			1.291	0.129		2.264	0.067		1.562	0.108
	TT	1.02±0.84		Ref	1.05±0.94		Ref	40.60±18.63		Ref
	TC	1.13±0.86		0.498	1.32±0.98		0.481	35.74±15.99		0.264
	CC	1.38±0.99		0.185	1.41±1.26		0.042	39.43±16.33		0.986

^a^ compared with ANOVA analysis, posthoc comparisons with SNK;

The significant results were in bold.

### Association between SNPs and PSQ Scores

In stage one, three SNPs (rs1541665, rs3772475, and rs757511) have been nominally found associated with PSQ scores (S5 Table). For rs1541665 and rs3772475, when comparing the hyperactive symptom score in the TT genotype group, the CC genotype showed only slightly higher value of the same score (*P* = 0.038 and *P* = 0.027, respectively). For rs757511, lower hyperactive symptom score has been found in AA genotype compared to that of the GG genotype. In validation stage, hyperactive symptom scores in CC genotype of rs1541665 and rs3772475 were found to be higher than the scores in the TT genotype (*P* = 0.029 and *P* = 0.046, respectively) (Table 4). Results have also been validated in combined samples. However, we didn’t find other snps significant association with PSQ scores (S5 Table).

### Gene-environment interactions

In the MDR analyses in combined samples, the three-factor model including rs1541665, maternal stress pregnancy and BLL was selected as the best predictor for ADHD risk, because it had the optimal testing accuracy and the maximal CVC values (Table 5). A significant interaction both in additive terms (*P*_add_ = 0.034) and multiplicative terms(*P*_mul_ = 0.021)was detectedwith an OR of 3.052 (95% CI, 2.219–4.271) among the subjects with maternalstress carrying an CT+CC genotype compared with those without maternalstress carrying TT genotypes (Table 6). Subjects with high lead exposure and CC+TC genotype exhibited significantly increased risk of ADHD compared tocarriers with low lead exposure and the wild-type genotypes, with ORs of 1.674 (95%CI = 1.299–2.478)on an multiplicativescale(*P*_add_ = 0.017).

**Table 5:** MDR analyses of the gene-environment interactions between SNP rs1541665 and ADHD risk factors in ADHD risk in combined samples.

Model	Testing accuracy	CV consistency	*P* for permutation*
Maternal stress pregnancy	0.6190	10/10	0.018
Maternal stress pregnancy, rs1541665	0.6448	10/10	0.009
**Maternal stress pregnancy, rs1541665, BLL**	**0.6468**	**10/10**	**0.001**
Maternal stress pregnancy, rs1541665, BLL, Low birth weight	0.6120	8/10	0.007

Abbreviation: BLL,Blood lead level; CV consistency, cross-validation consistency.

* The permutation test was carried out to repeat the MDR analyses 1000 times and calculate the CVC and testing accuracy of each model. The best model was in bold.

**Table 6:** Interaction analysis between environmental factors and polymorphisms associated with ADHD risk in combined samples.

Genotypes	factors	Case/control	OR(95%CI)	*P*^a^_mul_	*P*^a^_add_
rs1541665	Maternal stress pregnancy			**0.021**	**0.034**
TT	No	189/312	1.000		
TT	Yes	29/74	0.658(0.458,1.041)		
CT+CC	No	211/293	1.189(0.954,1.587)		
CT+CC	Yes	155/84	**3.052(2.219,4.271)**		
rs1541665	Blood lead level*				
TT	Low(<54.70ug/l)	148/272	1.000	0.154	**0.017**
TT	High(≥54.70 ug/l)	70/114	1.236(0.798,1.639)		
CT+CC	Low(<54.70ug/l)	132/141	1.731(1.263,2.471)		
CT+CC	High(≥54.70 ug/l)	234/276	**1.674(1.299,2.478)**		

*Blood lead level was divided into low and high by median (54.70 ug/l).

^a^ All the *P* values were adjusted for age and gender.

The significant results were in bold.

## Discussion

We conducted a two-stage case-control study to explore the effects of variants in Kv channels and their accessory proteins on the risk of ADHD, and identified *KChIP1* rs1541665 genetic variant as a new susceptibility locus for ADHD. The following analysis suggested that *KChIP1* rs1541665 also associated with hyperactive index score of PSQ scores and the ADHD-I risk. Gene-environmentinteractions analysis consistently revealed the potential interactionsof rs1541665 collaboratingwith maternal stress and blood lead to modify ADHD risk. To the best of our knowledge, this is the first study that comprehensively investigates the association betweenthe genetic variants of Kv channels and their accessory proteins and ADHD risk.

rs1541665, located in the intron of the *KChIP1* gene, was consistently shown to be associated with ADHD risk in the discovery and validation stages. *KCNIP1* gene (also known with its alias name *KChIP1*) encodes the potassium channel-interacting protein 1 (KChIP1), which is expressed predominantly in the brain, with relative abundance in cerebellum, hippocampus, striatum, and the reticular thalamic and medial habenular nuclei [43]. Abnormal function in cerebellum, hippocampus, and striatum has been known to be involved in ADHD risk [44]. KChIP1 was traditionally thought an auxiliary subunit of the protein complex Kv4.2 and Kv4.3 channels in neurons, and it has been reported to be able to dramatically increase the surface expression and slow the turnover of the Kv 4 protein, as well as increase the rate of recovery from the inactive state of Kv 4.2/4.3 channels, thereby reducing neuronal excitability [45, 46]. Indeed, evidence has shown that by reducing neuronal excitability through potassium channels regulation, KChIP1 plays a neuroprotective role against epilepsy [47]. Since high neuronal excitability has been associated to the pathogenesis of ADHD [48, 49], KChIP1 may be also be implicated in ADHD.

In addition, it may also be possible that KChIP1 involvement in ADHD is by regulating GABA levels, although the mechanism remains ambiguous. Studies have shown that KChIP1 potentially modulated GABAergic system by regulating inhibitory synaptic transmission [50, 51]. GABA is an inhibitory neurotransmitter and could play a role in ADHD. Courvoisie et al. measured decreased levels of GABA in ADHD patients [52]. An optimal level of GABA in the synaptic cleft maintains optimal excitability of the neuronal networks involved in alerting and attention [53, 54]. KChIP1 KO mice exhibited enhanced anxiety-like behavior related to GABA-mediated neurotransmission [51], and this anxiety-like behavior is a common trait in ADHD [55].

As a neuronal calcium sensor (NCS) protein, KChIP1 is also potentially involved in ADHD by regulating neuronal calcium. Calcium, which acts intracellularly like a second messenger, has an immediate impact on synaptic activity [56]. Gene disruption of neuronal calcium sensor -1 (NCS-1) causes defects in associative learning and memory, and methylphenidate alters its expression in the rat brain, suggesting a possible involvement of NCS-1 in ADHD [57]. In addition, other studies have reported that the activation of dopamine D2 receptors (D2R) suppressed backpropagating action potentials (bAP) controlled by KChIP1 [58] to evoke calcium transients most likely through the involvement of the dendritic Kv4 channels [59]. Given the vital role of D2R in ADHD [60, 61], KChIP1 has also been implicated in ADHD pathophysiology.

Gene-environment interaction analysis also showed KChIP1 rs1541665 collaboratingwith maternal stress pregnancy and blood lead to modify ADHD risk. Grizenko N et al. observed that maternal stress pregnancy may interact with the child’s DRD4 7/7 genotype to produce more severe ADHD symptoms [62]. The interaction of LPHN3 gene with maternal stress pregnancy showed to be associated with ADHD [63]. Plasticity or risk genotypes are likely susceptible to some riskfactors or stressors, supporting interaction of stress with genes [64]. Evidence has shown that adverse maternal environmentresulted in either hyper- or hypo-methylation for gene body and reduced expression of cell adhesion and neurotransmitter receptor genes, implicating a gene-environment interaction [65]. Lead has been as a risk factor for ADHD in previous reports [66, 67] and interacted with gene variants [68, 69]. Luo M et al. discovered the epigenetic mechanism bridging lead and ADHDin the histone modification level [70], and early life-lead exposure altered gene expression patterns and global methylation profiles, suggesting that lead caused neurotoxicity by gene interactions [71, 72]. Besides, lead can coordinate with the oxygen/sulfur atoms in the K channel protein, leading to the decrease in the function of the Kchannel [73]. Thus, we speculated that the lead and Kv channels may work together in ADHD. However, though we used the statistics methods to explore and find the potential interactions, the mechanism of how lead and maternal stress interact with KChIP1 remains unclear, which may be the limitations of our study. Further biological mechanism of the gene x environment interactions will be needed to identify the true interactions.

Neale et al found rs1541665 to be one of top 25 results associated with ADHD in corrected transmission disequilibrium test (TDT) (*P* = 5.60E-05) [10]. Although rs1541665 was found to have little biological function in F-SNP (http://compbio.cs.queensu.ca/F-SNP/) with an FS score of 0.101 for regulating transcription [36], the SNP was high LD with another 3’UTR SNP rs1363713 (D' = 1 and r^2^ = 0.905 in the 1000 Genomes Project, Phase 3 CHB), which was identified as a binding site for micro RNAs (miRNAs) by miRNA SNP2.0 (http://www.bioguo.org/miRNASNP/) [74]. The e-QTL analysis from the Brain eQTL Almanac (www.braineac.org) [75] indicated that rs1363713 genotype was associated with *KChIP1* gene expression (*P* = 0.0053 in substantia nigra). Accordingly, we could speculate that rs1541665 might not be the “real” causal variant for ADHD but only a “proxy” for other true biologically functional SNPs like rs1363713, which might provide a clue for further studies in the future.

In addition, in our study, we found evidence of *FHIT* rs3772475 and *KCNC1* rs757511 nominal association with ADHD and ADHD symptoms respectively, in the discovery stage. FHIT gene, encoding the fragile histidine triad protein, is preferentially and stably expressed in brain microglia. McCarthy et al. noted that FHIT gene was associated with ADHD by genomic study, in addition to having altered expression in the mouse brain in response to lithium [76]. KCNC1 encodes Kv3.1, a subtype of Kv channels, highly enriched in neurons firing at high-frequency [77]. KCNC1 also modulated gamma activity in neurons, which is disrupted in diseases associated with sensory processing and attention impairments [78, 79]. A reduction of gamma activity is observed in patients with Alzheimer’s disease, whereas an increase is found in patients with ADHD [80], implicating a role of the KCNC1 gene in ADHD.

In addition, in published GWAS studies, 5 loci (KChIP1 rs1541665, KChIP4 rs876477, DPP10 rs27200, FHIT rs3772475 and KCNC1 rs757511) were found to be associated with ADHD [10–12]. However, of these 5 loci, our study only found that KChIP1 rs1541665 was associated with ADHD, which is consistent with Neal’s GWAS findings. The inconsistent results of the other 4 loci may be attributed to the considerable differences in the allele frequencies of these SNPs between Asian and European descents. For example, the minor allele (the corresponding minor allele frequency, MAF) of KCNC1 rs757511 in Asian population and European population was A (0.31) and G (0.47) respectively.

In conclusion, this study comprehensively explored for the first time the role of Kv channels and their accessory proteins genes and their interaction with environment in ADHDand provided a clue for KChIP1 involvement in ADHD. Although we did not replicate the association in stage two for FHIT and KCNC1 and did not observe any association between other gene variants and ADHD,Neale et al and Stergiakouli et al. also did not show a clear effect for potassium channel genes [81, 82], which may be interpreted bythe inadequate power to detect effects, the differences of studied samples in geography, ethnicityand clinical phenotypes (persistence, comorbidity, etc.) and multiple genetic factors for complex disorders such as ADHD. Further studies with different ethnicities and further functional studies to reveal the biological mechanisms are warranted to produce definitive conclusions.

## Supporting information

S1 Table Characteristic data of the candidate polymorphisms. *MAF from 1000 Genomes Phase 3 CHB database.
(DOCX)

S2 Table Distribution of different genetic polymorphisms in ADHD and control group. Abbreviations: HW, wild type homozygote; HT, heterozygote; HV, variant homozygote; HWE: Hardy-Weinberg; MAF: minor allele frequency.
(DOCX)

S3 Table Association between individual SNP and ADHD risk in discovery stage. Abbreviations: Ref, Reference allele; HW, wild type homozygote; HT, heterozygote; HV, variant homozygote; OR, odds ratio; CI, confidence interval. a All the *P* values were adjusted for age and gender. The significant level was corrected with the formula of α' = α/15*5 = 0.00067 according to the Bonferroni method. The nominal significant results were in bold.
(DOCX)

S4 Table Correlations between candidate SNPs and ADHD subtype in stage one. ^a^ All the *P* values were adjusted for age and gender. The significant level was corrected with the formula of α' = α/15*3 = 0.0011according to the Bonferroni method. The nominal significant results were in bold.
(DOCX)

S5 Table Association of candidate SNPs genotypes with PSQ score in stage one. ^a^compared with ANOVA analysis, posthoc comparisons with SNK. The significant results were in bold.
(DOCX)
