# Supplementary material for: Attention-deficit/hyperactivity disorder associated with KChIP1 rs1541665 in Kv channels accessory proteins
Source: PLoS One. 2017 Nov 27;12(11):e0188678. doi: 10.1371/journal.pone.0188678 (PMC5703492; doi:10.1371/journal.pone.0188678)
Supplement: S1 Table — *MAF from 1000 Genomes Phase 3 CHB database. (DOCX) [file pone.0188678.s001.docx]

**S1 Table Characteristic data of the candidate polymorphisms**

| Gene | SNP | Chromosome | Position | Location | Minor/major allele | MAF* |
| --- | --- | --- | --- | --- | --- | --- |
| **KChIP4** | rs7668222 | 4p15.2 | 21858915 | 3’ UTR | T/C | 0.33 |
|  | rs876477 | 4p15.31 | 21156928 | Intron | T/C | 0.21 |
|  | rs4499696 | 4p15.31 | 21003851 | Intron | A/G | 0.30 |
| **KChIP1** | rs4868011 | 5q35.1 | 170099077 | Intron | A/C | 0.44 |
|  | rs2339091 | 5q35.1 | 169934412 | Intron | T/G | 0.47 |
|  | rs1541665 | 5q35.1 | 170142917 | Intron | C/T | 0.18 |
|  | rs4867981 | 5q35.1 | 169999591 | Intron | G/A | 0.36 |
| **DPP10** | rs2053724 | 2q14.1 | 116510817 | Exon | G/C | 0.26 |
|  | rs12472611 | 2q14.1 | 115964611 | Intron | C/A | 0.36 |
|  | rs10496492 | 2q14.1 | 116231075 | Intron | C/T | 0.32 |
|  | rs272000 | 2q14.1 | 116655795 | intergenic | G/C | 0.39 |
| **FHIT** | rs1825630 | 3p14.2 | 59902801 | Intron | C/T | 0.45 |
|  | rs4679478 | 3p14.2 | 60458639 | Intron | C/T | 0.41 |
|  | rs3821476 | 3p14.2 | 59935451 | Intron | T/C | 0.41 |
|  | rs717228 | 3p14.2 | 60602895 | Exon | T/C | 0.42 |
|  | rs3772475 | 3p14.2 | 59956549 | Intron | T/C | 0.38 |
| **KCNC1** | rs757511 | 11p15.1 | 17774473 | Intron | A/G | 0.26 |

*MAF from 1000 Genomes Phase 3 CHB database.
