# Supplementary material for: Attention-deficit/hyperactivity disorder associated with KChIP1 rs1541665 in Kv channels accessory proteins
Source: PLoS One. 2017 Nov 27;12(11):e0188678. doi: 10.1371/journal.pone.0188678 (PMC5703492; doi:10.1371/journal.pone.0188678)
Supplement: S2 Table — Abbreviations: HW, wild type homozygote; HT, heterozygote; HV, variant homozygote; HWE: Hardy-Weinberg; MAF: minor allele frequency. (DOCX) [file pone.0188678.s002.docx]

**S2 Table Distribution of different genetic polymorphisms in ADHD and control group**

| Gene | SNP | Minor allele | (HW/HT/HV) Frequency |  | χ2 | *P*^a^ | HWE | Call rate | |
| --- | --- | --- | --- | --- | --- | --- | --- | --- | --- |
|  |  |  | ADHD | Control |  |  | (*p* ^a^*value*) | (%) |  |
| KChIP4 | rs876477 | G | 90/127/39 | 133/197/39 | 3.085 | 0.214 | 0.006 | 99.5 |  |
|  | rs7668222 | T | 116/114/26 | 163/173/36 | 0.241 | 0.887 | 0.308 | 100.0 |  |
|  | rs4499696 | A | 134/87/25 | 189/144/39 | 0.838 | 0.658 | 0.145 | 98.4 |  |
| KChIP1 | rs2339091 | T | 95/108/48 | 134/178/58 | 1.988 | 0.370 | 0.930 | 98.9 |  |
|  | rs1541665 | C | 104/111/41 | 195/138/39 | 9.573 | 0.008 | 0.054 | 100.0 |  |
|  | rs4867981 | G | 116/107/30 | 166/157/49 | 0.256 | 0.880 | 0.222 | 99.5 |  |
|  | rs4868011 | C | 83/122/50 | 108/193/71 | 1.127 | 0.569 | 0.354 | 99.8 |  |
| DPP10 | rs272000 | T | 159/49/48 | 242/63/67 | 0.665 | 0.717 | 1.18*10^-27^ | 100.0 |  |
|  | rs10496492 | C | 79/130/45 | 99/187/80 | 2.134 | 0.344 | 0.638 | 98.7 |  |
|  | rs12472611 | C | 116/107/30 | 166/157/49 | 0.256 | 0.880 | 0.222 | 99.5 |  |
|  | rs2053724 | T | 93/118/43 | 129/158/82 | 2.666 | 0.264 | 0.013 | 99.2 |  |
| FHIT | rs3772475 | C | 114/106/36 | 205/128/39 | 6.958 | 0.031 | 0.007 | 100.0 |  |
|  | rs717228 | T | 83/122/50 | 108/193/71 | 1.344 | 0.511 | 0.354 | 99.8 |  |
|  | rs3821476 | G | 124/87/44 | 184/125/60 | 0.139 | 0.933 | 5.68*10^-6^ | 99.4 |  |
|  | rs1825630 | C | 85/117/50 | 129/182/61 | 1.236 | 0.539 | 0.812 | 99.4 |  |
|  | rs4679478 | C | 68/141/45 | 112/184/70 | 1.703 | 0.427 | 0.717 | 98.7 |  |
| KCNC1 | rs757511 | A | 104/113/37 | 143/156/71 | 2.249 | 0.325 | 0.021 | 99.4 |  |

Abbreviations: HW, wild type homozygote; HT, heterozygote; HV, variant homozygote; HWE: Hardy-Weinberg; MAF: minor allele frequency. The significant level was corrected with the formula of α' = α/17 = 0.003 according to the Bonferroni method.
