# Supplementary material for: Attention-deficit/hyperactivity disorder associated with KChIP1 rs1541665 in Kv channels accessory proteins
Source: PLoS One. 2017 Nov 27;12(11):e0188678. doi: 10.1371/journal.pone.0188678 (PMC5703492; doi:10.1371/journal.pone.0188678)
Supplement: S3 Table — Abbreviations: Ref, Reference allele; HW, wild type homozygote; HT, heterozygote; HV, variant homozygote; OR, odds ratio; CI, confidence interval. a All the P values were adjusted for age and gender. The significant level was corrected with the formula of α' = α/15*5 = 0.00067 according to the Bonferroni method. The nominal significant results were in bold. (DOCX) [file pone.0188678.s003.docx]

**S3 Table Association between individual SNP and ADHD risk in discovery stage**

| Gene | SNP | Ref | HTvsHW |  |  | HVvsHW |  |  | Dominant model |  |  | Recessive model |  |  | Addictive model |  |
| --- | --- | --- | --- | --- | --- | --- | --- | --- | --- | --- | --- | --- | --- | --- | --- | --- |
|  |  |  | OR（95%CI） | *P^a^* |  | OR（95%CI） | *P^a^* |  | OR（95%CI） | *P^a^* |  | OR（95%CI） | *P^a^* |  | OR（95%CI） | *P^a^* |
| KChIP4 | rs876477 | C | 0.954(0.673,1.390) | 0.547 |  | 1.440(0.886,2.405) | 0.480 |  | 1.128(0.673,1.890) | 0.647 |  | 0.424(0.037,4.929 | 0.493 |  | 1.073(0.660,1.743) | 0.776 |
|  | rs7668222 | C | 1.122(0.719,1.753) | 0.612 |  | 1.135(0.777,1.656) | 0.513 |  | 1.142(0.749,1.741) | 0.538 |  | 1.184(0.570,1.461) | 0.650 |  | 1.119(0.808,1.548) | 0.499 |
|  | rs4499696 | G | 1.185(0.755,1.860) | 0.460 |  | 1.111(0.818,1.508) | 0.502 |  | 1.194(1.790,0.805) | 0.399 |  | 1.124(0.633,1.994) | 0.691 |  | 1.121(0.842,1.494) | 0.434 |
| KChIP1 | rs2339091 | G | 1.096(0.667,1.802) | 0.717 |  | 1.066(0.794,1.431) | 0.669 |  | 1.124(0.710,1.781) | 0.617 |  | 1.081(0.651,1.793) | 0.764 |  | 1.075(0.804,1.439) | 0.635 |
|  | rs1541665 | T | 1.502(1.051,2.145) | 0.025 |  | 1.972(1.197,3.223) | 0.011 |  | 1.611(1.169,2.319) | 0.009 |  | 1.628(1.027,2.615) | 0.041 |  | 1.633(1.199,2.427) | 0.005 |
|  | rs4867981 | A | 1.041(0.659,1.644) | 0.864 |  | 1.006(0.699,1.447) | 0.975 |  | 1.033(0.668,1.598) | 0.884 |  | 1.033(0.523,1.043) | 0.925 |  | 1.026(0.740,1.423) | 0.877 |
|  | rs4868011 | A | 1.047(0.638,1.718) | 0.856 |  | 1.059(0.777,1.442) | 0.718 |  | 1.086(0.679,1.737) | 0.732 |  | 1.127(0.661,1.921) | 0.660 |  | 1.078(0.793,1.465) | 0.633 |
| DPP10 | rs10496492 | T | 0.928(0.584,1.475) | 0.753 |  | 0.977(0.710,1.345) | 0.887 |  | 0.925(0.597,1.433) | 0.727 |  | 0.958(0.542,1.694) | 0.882 |  | 0.952(0.702,1.289) | 0.748 |
|  | rs12472611 | A | 1.188(0.760,1.856) | 0.450 |  | 0.740(0.519,1.055) | 0.097 |  | 1.005(0.662,1.527) | 0.980 |  | 0.523(0.265,1.032) | 0.061 |  | 0.857(0.628,1.169) | 0.330 |
|  | rs2053724 | C | 0.783(0.497,1.234) | 0.292 |  | 1.040(0.709,1.527) | 0.839 |  | 0.840(0.550,1.284) | 0.421 |  | 1.151(0.550,1.410) | 0.708 |  | 0.929(0.673,1.282) | 0.655 |
| FHIT | rs3772475 | T | 1.517(1.194,2.195) | 0.024 |  | 1.662(1.001,2,515) | 0.049 |  | 1.572(1.128,2.306) | 0.011 |  | 1.459(0.823,2.341) | 0.173 |  | 1.557(0.910,2.537) | 0.165 |
|  | rs717228 | C | 1.019(0.623,1.668) | 0.939 |  | 1.030(0.762,1.393) | 0.846 |  | 1.020(0.643,1.618) | 0.932 |  | 1.026(0.613,1.718) | 0.921 |  | 1.017(0.757,1.366) | 0.912 |
|  | rs1825630 | G | 0.812(0.492,1.340) | 0.415 |  | 1.183(0.879,1.592) | 0.268 |  | 0.986(0.620,1.567) | 0.952 |  | 1.569(0.944,2.609) | 0.082 |  | 1.156(0.864,1.546) | 0.329 |
|  | rs4679478 | T | 1.326(0.812,1.164) | 0.260 |  | 1.126(0.827,1.533) | 0.453 |  | 1.318(0.828,2.098) | 0.245 |  | 1.094(0.645,1.857) | 0.738 |  | 1.161(0.855,1.577) | 0.339 |
| KCNC1 | rs757511 | G | 0.650(0.413,1.024) | 0.063 |  | 0.911(0.635,1.306) | 0.612 |  | 0.681(0.446,1.041) | 0.076 |  | 0.966(0.486,0.920) | 0.921 |  | 0.804(0.587,1.101) | 0.174 |

Abbreviations: Ref, Reference allele; HW, wild type homozygote; HT, heterozygote; HV, variant homozygote; OR, odds ratio; CI, confidence interval.

^a^ All the P values were adjusted for age and gender. The significant level was corrected with the formula of α' = α/15*5 = 0.00067 according to the Bonferroni method.

The nominal significant results were in bold.
