# Supplementary material for: Attention-deficit/hyperactivity disorder associated with KChIP1 rs1541665 in Kv channels accessory proteins
Source: PLoS One. 2017 Nov 27;12(11):e0188678. doi: 10.1371/journal.pone.0188678 (PMC5703492; doi:10.1371/journal.pone.0188678)
Supplement: S4 Table — a All the P values were adjusted for age and gender. The significant level was corrected with the formula of α' = α/15*3 = 0.0011according to the Bonferroni method. The nominal significant results were in bold. (DOCX) [file pone.0188678.s004.docx]

**S4 Table Correlations between candidate SNPs and ADHD subtype in stage one**

| SNP | Genotype | Control |  | ADHD-HI |  | |  | ADHD-I | | | |  |  | ADHD-C | |  | |  |
| --- | --- | --- | --- | --- | --- | --- | --- | --- | --- | --- | --- | --- | --- | --- | --- | --- | --- | --- |
|  |  |  | Case | OR（95%CI） | *P^a^* | | Case | OR（95%CI） | | | *P^a^* | | Case | OR（95%CI） | | *P^a^* | |  |
| rs876477 | CC | 133 | 20 | Ref |  | | 40 | Ref | | |  | | 30 | Ref | |  | |  |
|  | CT+TT | 236 | 44 | 1.185(0.687,2.694) | 0.475 | | 62 | 0.874(0.621,1.445) | | | 0.378 | | 60 | 1.116(0.531,2.817) | | 0.772 | |  |
| rs7668222 | CC | 163 | 30 | Ref |  | | 41 | Ref | | |  | | 45 | Ref | |  | |  |
|  | CT+TT | 209 | 34 | 1.193(0.614,2.309) | 0.612 | | 61 | 0.978(0.598,1.673) | | | 0.933 | | 45 | 1.297(0.715,2.359) | | 0.392 | |  |
| rs4499696 | GG | 189 | 30 | Ref |  | | 57 | Ref | | |  | | 47 | Ref | |  | |  |
|  | GA+AA | 183 | 34 | 1.138(0.621,2.085) | 0.674 | | 35 | 1.178(0.701,1.982) | | | 0.539 | | 43 | 1.467(0.816,2.631) | | 0.193 | |  |
| rs2339091 | GG | 134 | 15 | Ref |  | | 40 | | Ref | |  | | 40 | | Ref |  | |  |
|  | GT+TT | 236 | 49 | 1.228(0.765,2.812) | 0.253 | | 62 | 0.868(0.520,1.519) | | | 0.615 | | 50 | 1.623(0.724,3.389) | | 0.235 | |  |
| rs1541665 | TT | 195 | 30 | Ref |  | | 34 | | Ref | |  | | 40 | | Ref |  | |  |
|  | CT+CC | 177 | 34 | 1.018(0.375,1.778) | 0.612 | | 68 | | 2.089(1.401,3.137) | | 0.003 | | 50 | | 1.629(0.828,2.645) | 0.168 | |  |
| rs4867981 | AA | 166 | 30 | Ref |  | 47 | | | Ref | |  | | 39 | | Ref | |  |  |
|  | GA+GG | 206 | 34 | 1.492(0.873,3.176) | 0.231 | 55 | | | 0.924(0.628,1.539) | | 0.601 | | 51 | | 0.935(0.567,1.635) | 0.645 | |  |
| rs4868011 | AA | 108 | 20 | Ref |  | 35 | | | Ref | |  | | 28 | | Ref | |  |  |
|  | CA+CC | 264 | 44 | 1.014(0.459,2.275) | 0.910 | 67 | | | 1.102(0.659,1.648) | | 0.798 | | 62 | | 1.193(0.678,2.248) | 0.554 | | |
| rs10496492 | TT | 99 | 28 | Ref |  | 25 | | | Ref | |  | | 26 | | Ref | |  |  |
|  | CT+CC | 267 | 36 | 1.187(0.672,2.109) | 0.554 | 77 | | | 1.256(0.689,2.356) | | 0.558 | | 64 | | 0.578(2.394,1.165) | 0.067 | | |
| rs12472611 | AA | 166 | 30 | Ref |  | 48 | | | Ref | |  | | 38 | | Ref |  | | |
|  | CA+CC | 206 | 34 | 0.764(0.399,1.482) | 0.431 | 54 | | | 1.023(0.621,1.719) | | 0.968 | | 52 | | 1.321(0.725,2.378) | 0.379 | | |
| rs2053724 | CC | 129 | 25 | Ref |  | 38 | | | Ref | |  | | 30 | | Ref |  | | |
|  | CG+GG | 240 | 39 | 1.225(0.637,2.454) | 0.576 | 64 | | | 0.675(0.472,1.089) | | 0.101 | | 60 | | 1.031(0.596,1.853) | 0.912 | | |
| rs3772475 | TT | 205 | 32 | Ref |  | 45 | | | Ref | |  | | 37 | | Ref |  | | |
|  | TC+CC | 167 | 32 | 1.581(0.895,2.102) | 0.194 | 57 | | | 1.504(0.878,2.501) | | 0.128 | | 53 | | 1.891(1.008,2.881) | 0.044 | | |
| rs717228 | CC | 108 | 20 | Ref |  | 35 | | | Ref |  | | | 28 | | Ref |  | | |
|  | TC+TT | 264 | 44 | 0.868(0.520,1.819) | 0.715 | 67 | | | 0.978(0.598,1.773) | 0.943 | | | 62 | | 1.321(0.725,2.378) | 0.579 | | |
| rs1825630 | TT | 129 | 24 | Ref |  | 35 | | | Ref |  | | | 26 | | Ref |  | | |
|  | CT+CC | 243 | 40 | 0.928(0.420,1.819) | 0.824 | 67 | | | 0.967(0.538,1.713) | 0.843 | | | 64 | | 1.216(0.689,2.156) | 0.658 | | |
| rs4679478 | TT | 112 | 18 | Ref |  | 28 | | | Ref |  | | | 22 | | Ref |  | | |
|  | CT+CC | 254 | 46 | 1.221(0.725,2.578) | 0.679 | 74 | | | 1.594(0.878,2.511) | 0.134 | | | 68 | | 1.112(0.559,2.118) | 0.798 | | |
| rs757511 | GG | 143 | 23 | Ref |  | 47 | | | Ref |  | | | 34 | | Ref |  | | |
|  | GA+AA | 227 | 41 | 0.924(0.428,1.739) | 0.801 | 55 | | | 0.569(0.336,1.029) | 0.067 | | | 56 | | 0.731(0.512,1.298) | 0.276 | | |

^a^ All the P values were adjusted for age and gender.

The significant level was corrected with the formula of α' = α/15*3 = 0.0011according to the Bonferroni method.

The nominal significant results were in bold.
