# Supplementary material for: Attention-deficit/hyperactivity disorder associated with KChIP1 rs1541665 in Kv channels accessory proteins
Source: PLoS One. 2017 Nov 27;12(11):e0188678. doi: 10.1371/journal.pone.0188678 (PMC5703492; doi:10.1371/journal.pone.0188678)
Supplement: S5 Table — acompared with ANOVA analysis, posthoc comparisons with SNK. The significant results were in bold. (DOCX) [file pone.0188678.s005.docx]

**S5 Table Association of candidate SNPs genotypes with PSQ score in stage one**

|  |  | |  | | | | | | Conner score | | | | |  | | | |
| --- | --- | --- | --- | --- | --- | --- | --- | --- | --- | --- | --- | --- | --- | --- | --- | --- | --- |
|  | Genotype | | Impulsive-hyperactive score | | | | | | Hyperactive index score | | | | | Total score | | | |
|  |  | Mean±SD | | F | | | *P*^a^ | | Mean±SD | | F | *P*^a^ | | Mean±SD | F | | *P*^a^ |
| rs876477 |  |  | | | 0.340 | 0.281 | | |  | 0.798 | | | 0.184 |  | | 0.122 | 0.367 |
|  | CC | 1.16±0.68 | | |  | Ref | | | 1.17±0.42 |  | | | Ref | 39.17±18.28 | |  | Ref |
|  | CT | 1.26±0.64 | | |  | 0.926 | | | 1.31±0.52 |  | | | 0.476 | 38.00±17.79 | |  | 0.981 |
|  | TT | 1.27±0.63 | | |  | 0.881 | | | 1.30±0.48 |  | | | 0.615 | 38.36±15.57 | |  | 0.843 |
| rs7668222 |  |  | | | 4.871 | 0.013 | | |  | 4.638 | | | 0.016 |  | | 0.547 | 0.233 |
|  | CC | 1.24±0.46 | | |  | Ref | | | 1.12±0.64 |  | | | Ref | 37.39±17.65 | |  | Ref |
|  | CT | 1.25±0.43 | | |  | 1.000 | | | 1.25±0.57 |  | | | 0.727 | 39.44±15.81 | |  | 0.827 |
|  | TT | 1.40±0.55 | | |  | 0.230 | | | 1.34±0.53 |  | | | 0.328 | 42.50±21.38 | |  | 0.864 |
| rs4499696 |  |  | | | 0.240 | 0.312 | | |  | 0.798 | | | 0.187 |  | | 0.122 | 0.367 |
|  | GG | 1.16±0.68 | | |  | Ref | | | 1.15±0.42 |  | | | Ref | 39.17±18.28 | |  | Ref |
|  | GA | 1.26±0.64 | | |  | 0.926 | | | 1.31±0.52 |  | | | 0.476 | 38.00±17.79 | |  | 0.981 |
|  | AA | 1.17±0.63 | | |  | 1.000 | | | 1.30±0.44 |  | | | 0.605 | 37.36±14.57 | |  | 0.943 |
| rs2339091 |  |  | | | 0.401 | 0.268 | | |  | 0.292 | | | 0.298 |  | | 1.137 | 0.159 |
|  | GG | 1.22±0.65 | | |  | Ref | | | 1.36±1.11 |  | | | Ref | 38.25±18.34 | |  | Ref |
|  | GT | 1.31±0.56 | | |  | 0.880 | | | 1.34±0.48 |  | | | 0.999 | 40.33±16.97 | |  | 0.835 |
|  | TT | 1.15±0.54 | | |  | 0.854 | | | 1.18±0.44 |  | | | 0.620 | 32.65±10.21 | |  | 0.238 |
| rs1541665 |  |  | | | 1.672 | 0.099 | | |  | 3.266 | | | **0.035** |  | | 2.698 | 0.052 |
|  | TT | 1.28±0.48 | | |  | Ref | | 1.22±0.64 | |  | | | Ref | 36.35±14.51 | |  | Ref |
|  | CT | 1.30±0.46 | | |  | 0.974 | | 1.39±0.51 | |  | | | 0.260 | 39.43±16.19 | |  | 0.512 |
|  | CC | 1.58±0.63 | | |  | 0.136 | | 1.69±0.47 | |  | | | **0.038** | 48.60±24.87 | |  | 0.053 |
| rs4867981 |  |  | | | 0.247 | 0.312 | | |  | 1.022 | | | 0.159 |  | | 0.488 | 0.245 |
|  | AA | 1.17±0.61 | | |  | Ref | | | 1.30±0.39 |  | | | Ref | 39.47±18.33 | |  | Ref |
|  | GA | 1.26±0.69 | | |  | 0.894 | | | 1.28±0.56 |  | | | 0.481 | 36.92±16.75 | |  | 0.737 |
|  | GG | 1.24±0.69 | | |  | 0.997 | | | 1.34±0.55 |  | | | 0.792 | 38.87±12.81 | |  | 0.980 |
| rs4868011 |  |  | | | 0.501 | 0.239 | | |  | 0.192 | | | 0.334 |  | | 1.131 | 0.157 |
|  | AA | 1.23±0.65 | | |  | Ref | | | 1.36±1.11 |  | | | Ref | 38.25±18.34 | |  | Ref |
|  | CA | 1.31±0.55 | | |  | 0.880 | | | 1.34±0.48 |  | | | 0.999 | 40.33±16.97 | |  | 0.835 |
|  | CC | 1.05±0.54 | | |  | 0.855 | | | 1.10±0.42 |  | | | 0.620 | 35.65±10.21 | |  | 0.238 |
| rs10496492 |  |  | | | 1.291 | 0.129 | | |  | 2.264 | | | 0.067 |  | | 1.562 | 0.109 |
|  | TT | 1.38±0.64 | | |  | Ref | | | 1.35±0.46 |  | | | Ref | 37.60±18.63 | |  | Ref |
|  | CT | 1.23±0.62 | | |  | 0.197 | | | 1.42±0.43 |  | | | 0.481 | 35.74±15.99 | |  | 0.264 |
|  | CC | 1.28±0.59 | | |  | 0.185 | | | 1.61±0.46 |  | | | 0.092 | 39.43±16.33 | |  | 0.986 |
| rs12472611 |  |  | | | 0.243 | 0.312 | | |  | 0.791 | | | 0.184 |  | | 0.222 | 0.319 |
|  | AA | 1.16±0.68 | | |  | Ref | | | 1.15±0.42 |  | | | Ref | 39.17±18.28 | |  | Ref |
|  | CA | 1.26±0.64 | | |  | 0.922 | | | 1.31±0.52 |  | | | 0.476 | 38.03±17.79 | |  | 0.981 |
|  | CC | 1.17±0.63 | | |  | 1.000 | | | 1.30±0.44 |  | | | 0.605 | 37.36±14.57 | |  | 0.943 |
| rs2053724 |  |  | | | 0.561 | 0.229 | | |  | 0.292 | | | 0.298 |  | | 1.131 | 0.156 |
|  | CC | 1.23±0.65 | | |  | Ref | | | 1.36±1.11 |  | | | Ref | 38.25±18.34 | |  | Ref |
|  | CG | 1.31±0.55 | | |  | 0.880 | | | 1.34±0.48 |  | | | 0.899 | 40.03±16.94 | |  | 0.825 |
|  | GG | 1.25±0.54 | | |  | 0.895 | | | 1.20±0.42 |  | | | 0.620 | 31.65±10.21 | |  | 0.238 |
| rs3772475 |  |  | | | 3.127 | 0.041 | | |  | 4.639 | | | **0.016** |  | | 0.547 | 0.232 |
|  | TT | 1.24±0.46 | | |  | Ref | | | 1.12±0.45 |  | | | Ref | 37.29±17.66 | |  | Ref |
|  | TC | 1.26±0.47 | | |  | 0.923 | | | 1.25±0.58 |  | | | 0.727 | 39.41±15.82 | |  | 0.817 |
|  | CC | 1.61±0.55 | | |  | 0.042 | | | 1.52±0.53 |  | | | **0.027** | 42.51±21.37 | |  | 0.875 |
| rs717228 |  |  | | | 0.214 | 0.326 | | |  | 1.320 | | | 0.127 |  | | 0.473 | 0.251 |
|  | CC | 1.17±0.61 | | |  | Ref | | | 1.31±0.39 |  | | | Ref | 39.46±18.32 | |  | Ref |
|  | TC | 1.26±0.69 | | |  | 0.894 | | | 1.28±0.56 |  | | | 0.481 | 36.42±16.75 | |  | 0.737 |
|  | TT | 1.21±0.64 | | |  | 1.000 | | | 1.04±0.55 |  | | | 0.092 | 36.87±12.81 | |  | 0.882 |
| rs1825630 |  |  | | | 0.517 | 0.239 | | |  | 0.369 | | | 0.271 |  | | 0.799 | 0.187 |
|  | TT | 1.18±0.54 | | |  | Ref | | | 1.44±1.35 |  | | | Ref | 36.13±16.34 | |  | Ref |
|  | CT | 1.31±0.64 | | |  | 0.681 | | | 1.28±0.48 |  | | | 0.812 | 38.97±16.49 | |  | 0.678 |
|  | CC | 1.28±0.56 | | |  | 0.872 | | | 1.32±0.57 |  | | | 0.943 | 40.88±20.58 | |  | 0.637 |
| rs4679478 |  |  | | | 0.531 | 0.236 | | |  | 0.192 | | | 0.333 |  | | 1.131 | 0.159 |
|  | TT | 1.23±0.65 | | |  | Ref | | | 1.36±1.11 |  | | | Ref | 38.25±18.34 | |  | Ref |
|  | CT | 1.31±0.55 | | |  | 0.841 | | | 1.34±0.48 |  | | | 0.907 | 40.03±16.95 | |  | 0.825 |
|  | CC | 1.05±0.51 | | |  | 0.845 | | | 1.11±0.42 |  | | | 0.621 | 31.64±10.21 | |  | 0.338 |
| rs757511 |  |  | | | 0.842 | 0.179 | | |  | 3.201 | | | **0.037** |  | | 0.091 | 0.382 |
|  | GG | 1.11±0.65 | | |  | Ref | | | 1.43±0.33 |  | | | Ref | 38.00±16.11 | |  | Ref |
|  | GA | 1.32±0.57 | | |  | 0.479 | | | 1.33±0.51 |  | | | 0.129 | 37.78±18.96 | |  | 0.545 |
|  | AA | 1.19±0.68 | | |  | 0.973 | | | 1.19±0.46 |  | | | **0.038** | 39.51±15.63 | |  | 0.459 |

^a^ compared with ANOVA analysis, posthoc comparisons with SNK. The significant results were in bold.
